# Cell cyclins: triggering elements of cancer or not?

**DOI:** 10.1186/1477-7819-8-111

**Published:** 2010-12-22

**Authors:** Michael Stamatakos, Victoria Palla, Ioannis Karaiskos, Konstantinos Xiromeritis, Ioannis Alexiou, Ioannis Pateras, Konstantinos Kontzoglou

**Affiliations:** 1 4th Department of Surgery, Medical School, University of Athens, Attikon General Hospital, Athens, Greece; 2 1st Department of Surgery, Medical School, University of Athens, Laikon General Hospital, Athens, Greece; 3 Department of Vascular Surgery, Medical School, University of Athens, Attikon General Hospital, Athens, Greece; 4 2nd Department of Propaedeutic Surgery, Medical School, University of Athens, Laiko General Hospital, Athens, Greece

## Abstract

Cyclins are indispensable elements of the cell cycle and derangement of their function can lead to cancer formation. Recent studies have also revealed more mechanisms through which cyclins can express their oncogenic potential. This review focuses on the aberrant expression of G1/S cyclins and especially cyclin D and cyclin E; the pathways through which they lead to tumour formation and their involvement in different types of cancer. These elements indicate the mechanisms that could act as targets for cancer therapy.

## Introduction

Cyclins are proteins which act as key controlling elements of the eukaryotic cell cycle. These proteins have some regions of homology such as the cyclin box and some other islands of homology outside the cyclin box [1]. In mammalian cells, cyclins bind to cyclin dependent kinases and form complexes that are involved in regulating different cell cycle transitions: cyclin-D-CDK4/6 complex for G1 progression, cyclin- E - CDK2 for the G1-S transition, cyclin-A-CDK2 for S phase progression and cyclin A/B-CDC2 for entry into M-phase. In addition to these functions, cyclins are also involved in some processes not directly related to the cell cycle. The importance of cyclin-CDK complexes in cell proliferation is underscored by the fact that deregulation in the function of these complexes is found in virtually the whole spectrum of human tumors and this comes from the fact that tumor-associated alterations in cyclins help to sustain proliferation independently of external mitogenic or anti-mitogenic signals [2]. In this review we are going to deal with the role of cyclins D and E in the development of cancer, since these cyclins have proved to be of great importance for cancer pathogenesis.

## Cyclins and cell cycle

Considerable effort over many years has been expended in order to understand the mechanisms that control normal cell cycles. This effort has resulted in a detailed - but not yet completed - picture of the cell cycle revealing that complex oscillations in the activation and inactivation of cyclin- dependent kinase complexes propel mammalian cells through the cycle. The levels of most CDKs are relatively constant during the cell cycle but their activities depend highly on the state and level of activation of their cyclin partners or other regulatory molecules [3].

**Figure 1:**
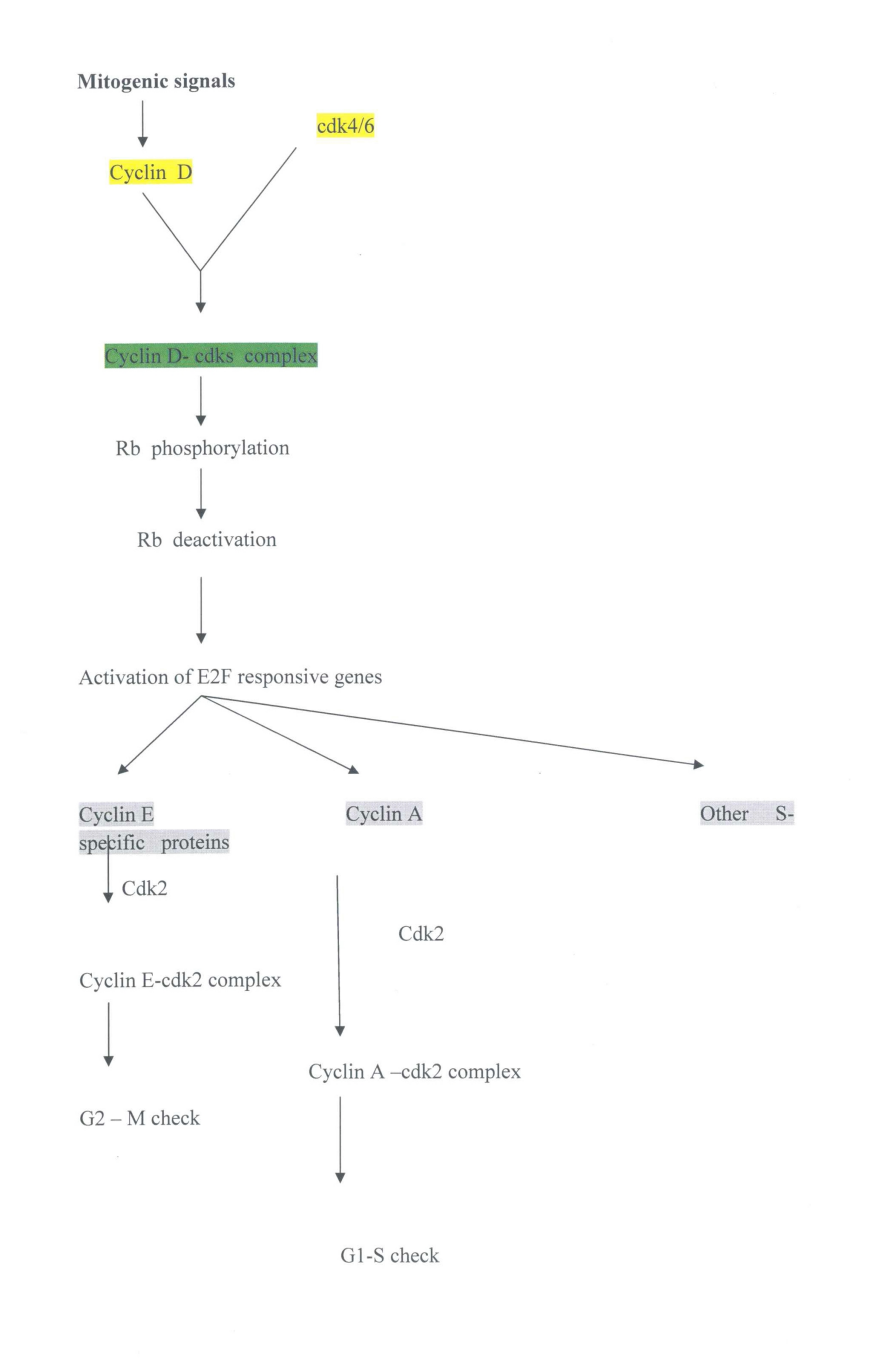
**Cyclins and cell cycle regulation**. This figure is a schematic presentation of the roleof cyclins in the cell cycle.

The triggering factor for progression to S phase is a mitogenic signal. In response to mitogenic activation, cells synthesize D-type cyclins which form a holoenzyme with CDK4, CDK6. Cyclin D1 is the regulatory subunit whereas the CDKs are the catalytic subunit (figure 1). This assembly of proteins needs members of the Cip/Kip families of proteins which promote the activity of cyclin D dependent kinases and serve as inhibitors of CDK2. [4]. The active complex phosphorylates the pRB protein and leads to its inactivation. The inactivated pRB protein seperates from the complex of pRB and E2F transcription factors giving permission to genes required for S phase to be transcripted [3]. Cyclin E, cyclin A and DNA pol stand among these genes. Cyclin E binds to CDK2 leading to phosphorylation of substrates required for proper replication firing, centrosome duplication and histone biosynthesis [5]. Cyclin E and its partner, CDK2, can also further phosphorylate and inactivate pRB. Cyclin A binds to CDK2 and this complex phosphorylates CDC6 resulting in its relocalisation from the nucleus to the cytoplasm and in this way to its destruction. This procedure prevents CDC6 from assembling into origins of replication of DNA after G1. DNA re- replication is also avoided by the procedure where cyclin A -CDK2 phosphorylates MCM4 in the helicase complex and eventually inhibits its DNA helicase activity [6].

To summarize, such complicated, multilevel controls on expression and activation of cyclin/CDK complexes permit exquisite and necessary coordination of the cell cycle stages and thereby prevent from the formation of tumor cells [2].

## Cyclin D and cancer

Cyclin D is solidly established as an oncogene with an important pathogenetic role in many human tumors. There are three highly homologous and almost indistinguishable biochemically D- type cyclins (D1, D2 and D3) in mammalian cells which are binded to either CDK4 or CDK6 in a tissue specific way. Among these types, cyclin D1 is the one most commonly expressed in several human cancers [6]. Cyclin D1 is a 35-kDa protein which is encoded by 5 exons situated at the region of chromosome band 11q13. In the aminoterminus of cyclin D1 appears a motif Leu - X - Cys - X - Glu (X represents any aminoacid) where pRB pocket domain binds. The carboxy terminus inhibits myogenic helix loop helix (HLH) protein function. HLH protein main action is to remove cells from the cell cell cycle (halt proliferation), so its inhibition by cyclin D1 leads the cell to G1 stage of the cell cycle. Repression by D cyclins appears to be independent of its effects on the cell cycle [7]. The protein is quite unstable with a half - life of less than 20 minutes; its degradation is ubiquitin proteosome- regulated [8].

Cyclin D1 is overexpressed in several human tumours. Chromosomal translocations, gene amplification and disruption of normal intercellular trafficking and proteolysis are the procedures which lead to accumulation of cyclin D1 in tumor cell nuclei and eventually to cyclin D1 overexpression in many tumours.

Chromosomal translocations are very common among parathyroid adenomas, B mantle cell lymphomas and multiple myelomas. Gene amplification (11q13) as a mechanism for aberrant overexpression of cyclin D1 is associated with non- small cell lung cancers, head and neck squamous cell carcinomas, pancreatic carcinomas, bladder cancer, pituitary adenomas and breast carcinoma. Emerging evidence suggests that nuclear retention of cyclin D1 resulting from altered nuclear trafficking and proteolysis is critical for the manifestation of its oncogenicity [9]. Disruption of the normal intracellular trafficking and proteolysis of the nuclear non - phosphorylatable cyclin results from a polymorphism in exon four of cyclin D1. This leads to a C-terminus that lacks the phosphor- acceptor site that targets cyclin D1 for cytoplasmic destruction [10].

Cyclin D oncogenic potential is manifested in several ways. As mentioned before, it leads to direct activation of CDK4/6. In addition to this function, cyclin D1/CDK complexes bind and sequester p21CIP1 and p27KIP1, which among others function as inhibitors of cyclin E/CDK2. [11]. In this way, both high expression of cyclin D1 and deregulated expression of cyclin E1 cooperate to increase tumour fitness. Another cyclin D1 function that can lead to tumour formation is the transcriptional control that does not involve CDKs. This function involves promoter recruitment of histone deacetylases (HDACs) and histone methyltransferases. Normally HDAC, by increasing the positive charge of histone tails and histone methylotransferases, through the methylation of histones, can both lead to high- affinity binding between histones and DNA backbone. In this way, DNA structure condenses and transcription is prevented [12].

Several groups have demonstrated that cyclin D1 can also act as a transcriptional co-factor for steroid hormone receptors such as estrogen receptor [13].

Besides tumour formation, cyclin D1 can also play a pivotal role in the invasiveness and the metastatic phenotype through the interactions between the malignant cell and the host environment. For example, overexpression of cyclin D1 through the activation of positive feedback loop of E2F-1 mediated transcription can lead to excessive expression of FGFR-1 (fibroblast growth factor receptor 1) [14]. FGFR up - regulation has been shown in several tumours such as brain, breast, prostate, thyroid, skin and salivary gland tumours. Additionally, cyclin D1 normally plays a regulatory role in angiogenesis and mithochondrial function. This suggests that deregulated cyclin D1 expression can contribute to the invasive and metastatic potential of a tumour, since mtDNA mutations can lead to development of metastases by overproduction of reactive oxygen species (ROS) [15,16].

The biological importance of these functions needs to be proved *in vivo*; nevertheless it is obvious in concept that they could be of variable impact on tumour phenotype. Nevertheless, solitary cyclin overexpression is not sufficient for malignancy transformation. Additional cellular abnormalities are necessary for the tumour formation [17].

Table 1 describes the way that cyclin D is associated with several types of cancer.

**Table 1:** The role of cyclin D in several types of cancers

Parathyroid adenomas	Cyclin D overexpression in both neoplastic and non- neoplastic proliferating parathyroid tissue.
Papillary thyroid carcinoma	lymph node metastasis

Mantle cell lymphoma	Higher age distribution Larger cell size Higher mitotic index More frequent gastrointestinal involvement Higher international prognostic index score Lower p27 expression Significantly worse survival

breast cancer	higher tumour grade no correlation with axillary lymph node status or tumour size or HER2 amplification poorer prognosis indication for need for additional chemotherapeutic treatment positive correlation with ER status (p < 0.005) positive correlation with PR status (p < 0.005) inverse correlation to Nottingham prognostic index inverse correlation to membrane EGFR significantly shorter overall survival and relapse - free survival tamoxifen resistance

Parathyroid adenomas are a common disease where cyclin D1 is overexpressed. The pericentromeric inversion of chromosome 11 places the 5' regulatory region of the PTH gene on 11p15 immediately upstream of cyclin D1 gene promoter. Many studies have taken place and they have demonstrated a cyclin D1 overexpression which varies between 20 - 40% [18]. Nevertheless, overexpression of cyclin D1 is also found in nonneoplastic proliferation of parathyroid gland, but not in the normal parathyroid tissue. The hormonal regulatory defect in parathyroid adenomas can be both primary and secondary to a defect in cellular - growth control indicated by cyclin D1 oncogene overexpression [19].

Papillary thyroid carcinoma is another malignant tumour where cyclin D1 is overexpressed. In addition to this, the level of cyclin D1 expression according to lymph node metastasis was statistically significant (P < 0.05). This fact indicates that cyclin D1 may be a useful marker for the evaluation of lymph node metastasis

In addition to solid tumours, overexpression of cyclin D1 has also been reported in certain lymphoid malignancies. Referring to B- cell non Hodgkin lymphomas, cyclin D1 was mainly overexpressed in mantle cell lymphomas and large B- cell lymphomas whereas the other subtypes showed normal cyclin D1 expression. Clinical signs (except for lymphadenopathy) and laboratory data (except for LDH) were not influenced by cyclin D1 overexpression which, nevertheless, proved to be associated with poor outcome of NHL patients [20]. More specifically, mantle cell lymphoma, which accounts for 5 - 10% of all non Hodgkin lymphomas, demonstrate chromosome translocations (t(11;14)) involving the immunoglobulin heavy chain IgH locus that lead to cyclin D1 deregulation [21]. Mantle cell lymphomas express variable levels of cyclin D1 at both transcript and protein levels. Overexpression of cyclin D2 and D3 has also been described. As far as mRNA forms produced by cyclin D1 gene (long - D1L, short - D1S) are concerned, the short version has been shown to be more related to blastoid histology than the long version (60% of D1 S and 9% of D1L) [22]. In addition to these notifications, cyclin D1 was also identified as a potentially important antigen for immunotherapy of mantle cell lymphoma as it was proved to be recognized by potent cytotoxic T cells when it was naturally presented by lymphoma cells in the context of HLA - A * 0201 molecules [23]. Another subtype of non Hodgkin lymphomas, known as diffuse large B - cell lymphoma, shows overexpression of D1 (2%) D2 (49%) and D3 (20%) cyclins [24]. A small subset of chronic lymphocytic leukemias overexpresses cyclin D1 in amounts that can be demonstrated by immunohistochemistry [25].

Cyclin D1 is solidly established as an oncogene with a pivotal role in pathogenesis of breast cancer. Besides gene amplification, cytoplasmic sequestration may also serve to regulate cyclin D1 activity in mammalian cancer cells [26]. Emerging evidence indicates that cyclin D1 may act, in part, through pathways which do not involve its role as a cell cycle regulator. One such function is the cyclin D1 contribution to cell adhesion and motility. So, it was demonstrated that cyclin D1/CDK4 complex interacts with filamin A (member of the actin - binding filamin protein family) and influences the migration and invasion potential of breast cancer cells [27]. CCDN1 amplification is found in 5 - 20% of primary breast cancers [28].

## Cyclin E and cancer

Human cyclin E cDNA was identified in 1991 by screening human cDNA libraries for genes that could complement G1 cyclin mutations in yeast S. cerevisiae [29]. Cyclin E is derived from a gene on chromosome 19q12 → q13. This gene encodes a variety of polypeptides with molecular weights ranging from 39 to 52 kDa. The "regular" form contains the "cyclin box", a sequence set in amino acid position 129-215, which is partly common among the cyclins. In addition to the regular form, two splice variants and an isoform with 15 additional aminoacids at the N-terminus have also been described [30]. More recently six splice variants with the potential to produce cyclin E isoforms of substantially altered molecular weight have been found [31]. All splice variants with an intact cyclin box have the ability of binding and activation of CDK2 [32].

The deregulated expression and activity of cyclin E have been associated with a variety of cancers and it is considered to be involved in the oncogenic process [33]. The oncogenic activity of cyclin E is a result of several mechanisms. Cell cycle deregulation of cyclin E expression is common in some tumour cells leading to constitutive cyclin E expression and activity throughout the cell cycle. Overexpression of cyclin E can come from gene amplification in most cases [34]. For example cyclin E gene is amplified by 8 fold and its mRNA is overexpressed by 64 fold in a subset of breast cancer cell lines [35,36]. Defected degradation via the proteosome is another mechanism leading to cyclin E overexpression; the F- box proteins that target cyclin E for ubiquitination and as a result for degradation were discovered to be mutated in some cancers [37]. Cyclin E overexpression can lead to G1 shortening, decrease in cell size or loss of serum requirement for proliferation. This is the consequence of cyclin E normal function of S - phase induction; one of the pathways involving pRB has already been described; in addition cyclin E/CDK2 complexes have been proved to activate transcriptional regulators like human B - MYB and NPAT which are of great importance for cellular proliferation [38]. Besides the mechanisms already described, cyclin E demonstrates its oncogenic potential by a correlation with oncogenic viruses. HPV and especially HPV E7 protein which is implicated in cervix carcinoma, can lead to promotion of cyclin E - associated kinase activities through the interaction with p21 (which is a cyclin - dependent kinase inhibitor) [39]. CMV has a dual role in activating cyclin E through direct induction of cyclin E and inactivation of cyclin - dependent kinase inhibitors [40]. On the other hand, HIV - 1 halts cyclin E activity and causes G1 phase arrest, which encourages viral replication [41].

Cyclin E is a factor found in a variety of cancers like breast, ovarian, colorectal, bladder and other. In Table 2 the way cyclin E is associated with several types of cancer is depicted.

**Table 2:** Cyclin E and its role in different types of cancer

Breast cancer	poor disease free survival Poor overall survival High tumor grade High tumor stage Lack of steroid receptors HER - 2/neu expression Ki - 67 expression BRCA1 germline mutations Triple negative breast tumours Basal - type keratins (CK 5/6 or CK17) expression Bone, visceral and in general distant relapse
Ovarian Cancer	Controversial correlation with prognosis Serous, clear cell and poorly differentiated carcinomas Higher tumour grade Late stage disease Patient age more than 60 years old at the time of diagnosis Suboptimal cytoreduction Controversial correlation with lifetime ovulatory cycles (LOC) No correlation with the chemotherapy response Marker of aggressive disease in patients with metastatic ovarian carcinoma (low molecular weight isoforms)

Gastric cancer	Promotion of the progression of early gastric cancer Prediction of the survival in early - onset gastric cancer (LMW isoforms) Poor histological grade Serosa invasion Advanced stage Tumour size (p > 0.05) Lymphatic invasion CDK - 2 expression pRb expression

Colorectal carcinomas	Increased risk of recurrence Worse outcome Possible prognostic marker in non metastatic colon cancer Correlation with p21 waf1/cip1 and cell proliferation Blood vessel invasion Gross configuration of the tumour Independent prognostic factor in rectal carcinoma at stage I - III

Melanomas	Histological type Tumour stage Significant association with some specific tumour subtypes

Non small cell lung carcinoma	Poorer survival among stage I to IIIa Invasion of local structures Poor prognosis Ki - 67 labeling index Distant metastases

With regard to breast cancer, a remarkable number of studies have been driven. Altered expression of cyclin E occurs in 18 - 22% of the breast cancers and can serve as potential prognostic marker [42]. The expression of low molecular weight cyclin E derivatives has been investigated with great emphasis since they have been shown to be of great pathogenetic and prognostic importance for breast cancer. Low molecular weight isoforms are resistant to CKIs, bind more efficiently to CDK2 and can stimulate the cells to progress through the cell cycle more efficiently [43,44]. As a result resistance to anti - growth signals and genomic instability are more common. These forms have proved to be a remarkable marker of the prognosis of early -stage - node negative breast carcinoma [45].

Cyclin E has also been correlated with ovarian carcinomas, the fourth leading cause of cancer deaths among women in the United States. In ovarian carcinomas, cyclin E is overexpressed primarily in the low molecular weight isoforms [46] which are both biochemically and biologically hyperactive as mentioned before. The exact correlation between cyclin E overexpression and prognosis is controversial.

Cyclin E overexpression is also implicated in carcinomas at various sites along the gastrointestinal tract, but the most important sites are the stomach and the colorectal region [47]. As far as stomach cancers are concerned, cyclin E overexpression was found in 50 - 60% of gastric adenomas and adenocarcinomas [48]. Cyclin E was shown to be of independent prognostic significance in gastric carcinoma [49].

Regarding the colorectal carcinomas, cyclin E gene amplification is quite rare, estimated at the level of 10% [50]. Overexpression of cyclin E is detected in the early stages of the carcinogenic process promoting the morphological progression from adenoma to adenocarcinoma and the progression of early cancer [50,51]. Cyclin E was detected in both full length and low molecular weight forms in tumour and adjacent macroscopically normal mucosa.

Cyclin E overexpression has also been reported in melanomas. Cyclin E, in combination with other cell cycle regulators, has been proved to be of determinant significance for melanoma growth and/or transformation and this is indicared by the fact that cyclin E was not detected in benign naevi but it was easily detectable in most of the metastatic melanomas [52]. Another study has explicated the importance of the low molecular weight isoforms of cyclin E in melanoma formation. It was found that the isoforms were overexpressed in a subset of primary invasive and in metastatic melanomas but not in benign naevi. The low molecular weight forms are histologically active and can function as regulators of invasion and metastasis since they can form angiogenic tumors with prominent perineural invasion and increase the incidence of metastases in comparison to the full - length cyclin E [53].

Implication of cyclin E in the non small cell lung carcinomas has been the subject of several studies. The association with deeply invasive tumours and the function of cyclin E as an independent factor for poor prognosis were also proved by another study [54].

## Conclusions

In conclusion, cyclins play a multifunctional and pivotal role in the pathogenesis of cancer. This is the reason why alterations in their structure and function through the influence of various pathways can lead to an array of cancer types. This discovery in combination with recent studies in genetically engineered mouse models implies their potential role in cancer therapy and especially targeted therapies. Despite the clinical applications of cell cycle specific chemotherapeutic agents there is still urgent need to develop novel drugs that are able to target multiple sites and pathways of the cell cycle [55].

## Competing interests

The authors declare that they have no competing interests.

## Authors' contributions

MS: partial English editing and correction, VP: partial English editing, IK: search of the literature, KX: editing and correction, IA: search of the literature, IP: search of the literature, KK: final editing and corrections. All authors read and approved the final manuscript.
